# Using ANPR data to create an anonymized linked open dataset on urban bustle

**DOI:** 10.1186/s12544-022-00538-1

**Published:** 2022-04-24

**Authors:** Brecht Van de Vyvere, Pieter Colpaert

**Affiliations:** 1 Ghent University, Technologiepark-Zwijnaarde 122, Ghent, 9052 Belgium; 2 Department of Electronics and Information Systems, IDLab Ghent University - imec, Ghent, Belgium

**Keywords:** ANPR cameras, Anonymization, Semantic interoperability, Smart cities, Digital twin

## Abstract

ANPR cameras allow the automatic detection of vehicle license plates and are increasingly used for law enforcement. However, also statistical data generated by ANPR cameras are a potential source of urban insights. In order for this data to reach its full potential for policy-making, we research how this data can be shared in digital twins, with researchers, for a diverse set of machine learning models, and even Open Data portals. This article’s key objective is to find a way to anonymize and aggregate ANPR data in a way that it still can provide useful visualizations for local decision making. We introduce an approach to aggregate the data with geotemporal binning and publish it by combining nine existing data specifications. We implemented the approach for the city of Kortrijk (Belgium) with 43 ANPR cameras, developed the ANPR Metrics tool to generate the statistical data and dashboards on top of the data, and tested whether mobility experts from the city could deduct valuable insights. We present a couple of insights that were found as a result, as a proof that anonymized ANPR data complements their currently used traffic analysis tools, providing a valuable source for data-driven policy-making.

## Introduction

The implementation of Automatic Number Plate Recognition (ANPR) cameras has increased enormously in Flanders (the northern part of Belgium). The province of West Flanders went from a dozen cameras in 2012 to 226 in 2020 [1], creating a vast sensor network of surveillance cameras [2]. The collected data from these cameras include the time, location and license plate of detected vehicles and are therefore Personally Identifiable Information (PII). On the one hand, these data are used in a secured environment for law enforcement or investigation of travelling criminals. On the other hand, cities could use the data for better policy-making, but they need to balance transparency requirements with privacy best practices [3, 4]. This balance is already being investigated in the context of shared mobility [4, 5]. However, to the best of our knowledge, there is no related work found proposing an approach to generate anonymized ANPR data that is still useful for local decision making.

A network of ANPR cameras can become an important data source for digital twin infrastructure. Digital Twins (DT) enable comprehensive data exchange and can contain models, simulations, and algorithms describing their counterparts, including their features and behavior in the real world [6]. Related work has shown that ANPR data provide a rich, insightful source of information about travel behaviour [7–9]. However, the authors didn’t incorporate an anonymization approach thus it is still unclear which anonymization techniques should be applied to make ANPR data shareable and reusable for policy-making. Privacy-sensitive data cannot be shared outside its protected environment without authentication and authorization mechanisms. Even then it is questionable whether a local authority should build applications on top of the raw, unprocessed data. In the state of the art of shared mobility and traffic counting devices [4, 5, 10], anonymization techniques are fruitfully applied to create publicly available datasets and dashboards. This way, local authorities can be transparent with the kinds of data they collect [4] and stimulate its reusability [3].

Aligning the anonymized data with semantic data specifications is another aspect that this study provides new insights into [11]. Semantic data specifications are a key aspect of a comprehensive data exchange with a DT [12, 13]. For the European Commission [14], semantic interoperability means that organizations can process information from external sources in a meaningful manner. It ensures that the precise meaning of exchanged information is understood and preserved throughout exchanges between parties. Previous work of Ivanov et al. [2] only mentioned data cleansing as a component of a digital twin information infrastructure. They did not deal with a component to perform a semantic uplift of data sources in order to allow infrastructure components to process this data more easily and become reusable.

The purpose of this study is to (i) investigate how ANPR data can be anonymized based on the state of the art, (ii) explore which existing semantic data specifications can be used, (iii) demonstrate how visualizations can be created from these semantic data, and (iv) validate with data from a city whether the generated visualizations still contribute to policy-making. This study is not only of interest for authorities that have access to a set of ANPR cameras, but also organizations that use traffic counting devices in general and want to share their data in a semantically interoperable format. With the open source ANPR Metrics tool proposed in this study, these organizations will also be able to reproduce our approach for their datasets. The rest of this study comprises five sections. First, we provide background on anonymizing with k-anonymity and semantic data. Then, we describe similar work on anonymizing data in the mobility domain. In the method section, the anonymization process and semantic description of the anonymized data is explained. Then, this process is applied in a case study with the city of Kortrijk. Finally, we will discuss how these results set the first step towards publishing anonymized ANPR data as a valuable source for data-driven policy-making.

## k-anonymity and semantic data

In this section, we first describe how anonymizing data with k-anonimity works. The exchange of these anonymized data is best done on the basis of a standard to uniformly describe data independently of the various types of ANPR cameras and their software. Therefore, we will also describe how semantic data are a key mechanism for interoperability between data providers.

*k-anonymity* is an anonymization technique [15–17] that has been widely used in releasing datasets from databases preventing identity disclosure based on the quasi-identifiers [18]. Previous studies mostly defined “quasi-identifiers” as subsets of attributes that indirectly identify an individual (e.g. gender, zip code, location/time pair) [19]. k-anonimity requires that each record should be equivalent with at least $k-1$ other records in order that there is 1/*k* chance of attributing a row to an individual. Some studies [18] differentiate between quasi-identifier attributes and confidential attributes and extend k-anonimity (*t*-closeness, *l*-diversity) to control the variability of the confidential attributes. However, there is a risk of attribute disclosure as Soria-Comas [18] notes: ‘if the values for one (or several) confidential attribute(s) are identical within a group of records sharing the quasi-identifier attribute values, attribute disclosure happens from a dataset.’ In the context of ANPR data, a license plate of a detected vehicle can be categorized as confidential attribute and the location/time pair as quasi-identifier attribute. Finally, there are two basic approaches currently being adopted into k-anonymity. One is generalization where a given attribute is replaced with more general values, and another is suppression, which prevents the release of an attribute.

*Semantic data* The term ‘semantic data’ is used to refer to data that are structured with the Resource Description Framework (RDF). RDF is a standard data model used for the exchange of data on the Web and uses statements in the form of a triple ($$<subject><predicate><object>$$ ) to express information about resources [20–22]. A resource can be various things, such as an ANPR camera, the observation created by software tooling, or an entity classification. The subject and object are related resources with the predicate element describing its relation. All three elements can be globally identified with a Web address, called Uniform Resource Identifier (URI). When the URI of a resource can be dereferenced, machines are able to look up more information. A dataset becomes interlinked when referring to URIs of other datasets. For example, an inventory of ANPR cameras can be interlinked with a road registry when the cameras are linked with the observed road segments: $$<ANPR camera><observes road segment><road segment>$$. The interlinked descriptions of entities with RDF lead to the creation of a knowledge graph [23] visualized as a directed labeled graph in which the labels have well-defined meanings [24]. Next to reusing URIs for the subject and object resources, predicates also need to be defined globally with URIs allowing machines to retrieve the predicate’s machine-readable description. Multiple standardization bodies exist on different levels (W3C [25], ISA [21], OSLO [26], OASC [27], ETSI [28]) governing semantic specifications stating which URIs should be used for common relation and classification types and provide their formal descriptions. This way, semantic data specifications are a crucial mechanism to ensure interoperability between different verticals and actors in smart cities to share and receive similar information and, in addition, reduce the integration cost for technology vendors to roll out their products in multiple cities.

## State of k-anonimity in mobility services

This section will introduce two approaches for anonymizing trip data of shared mobility services, and one approach for anonymizing traffic counts. The first two use data coming from Application Programming Interfaces (APIs) aligned with the Mobility Data Specification (MDS) [29]. MDS allows dockless, shared mobility services, such as e-scooters, bicycles, mopeds and carshare, to inform cities, for example, where and when vehicles are dropped off or performed a trip. Conversely, the city can also set up an API to communicate regulation, such as which zones are forbidden for these dockless vehicles. The term ‘binning’ is used here to refer to grouping or aggregating data points in a certain dimension.

**Fig. 1:**
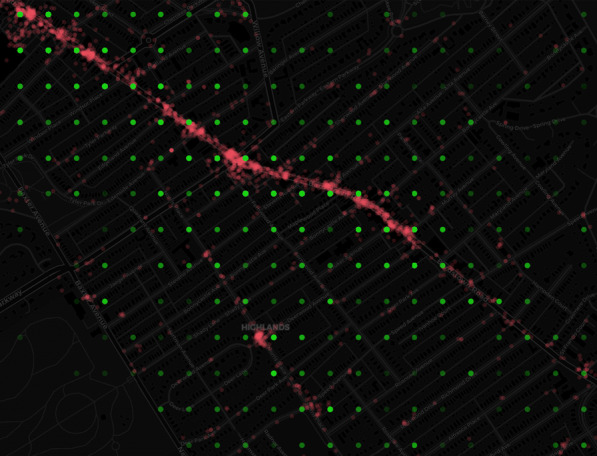
Raw GPS (red) versus binned (green) departure and arrival location of a trip

*Dockless open data* This specification is created by the Chief Data Officer of Louisville (US) [4] to anonymize trip data originating from an MDS data source. It describes a set of Structured Query Language (SQL) commands to generate an anonymized dataset that can be published as open data. This dataset allows cities to visualize the dockless vehicles’ demand, and more specifically, which locations are popular pick-up and drop-off locations. This way, cities can better answer infrastructure needs, such as where bicycle parking lots are needed. In Fig. 1, we can see these locations indicated with red dots. However, they are obtained from the raw GPS locations of vehicles. Note that only the start and end points of a trip are used to make an origin/destination (O/D) pair. To protect a rider’s privacy and achieve transparent policy-making, Dockless Open Data defines three steps to create an anonymized subset of the trip data (displayed in Fig. 1 with green dots).

1. Time binning: the start and end timestamps of a trip are grouped into bins of 15 minutes.
2. Geographic binning: the precision of the start and end location of an O/D pair is set to three decimal places. This rounding can be visualized with a grid where locations are grouped into dots with a distance of 100 x 80 m.
3. k-anonymity generalization with geographic fuzzing: when a group of O/D pairs with the same start and end location contains less than five pairs (k=4), then their start and end locations are randomly moved to another grid point within a 400-meter radius around its grid point of step 2.

Dockless vehicles can have a wide coverage in a city: in Louisville, one-third of the O/D pairs still need to be geographically fuzzed to fulfill the requirement of minimally 5 O/D pairs per grid point. In the second step, location points can be moved 1 to 100 meters away from their original location. In the third step, this moved location point can again be moved within a circle with a radius of up to 400 meters in any direction, meaning that the original location point is located within a circle of 800 meters diameter around the moved location point. The effect of this approach is that sparse bins are fuzzed away by joining dense bins. As a result, the derived dataset is still useful for the analysis of the most frequently used areas, because the integrity of the derived dataset is reasonably maintained [4].

**Fig. 2:**
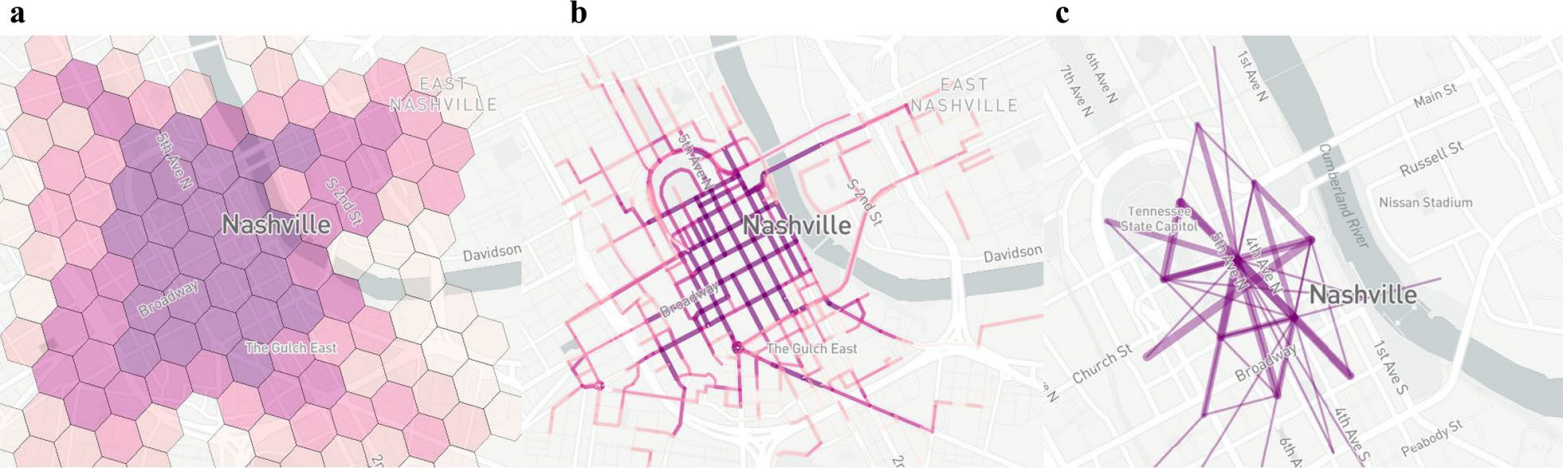
Geographic binning performed per H3 hexagon (**a**), road segment (**b**), and origin/destination pair between H3 hexagon’s centroids (**c**)

*Mobility metrics* This tool [5] provides more features than Dockless Open Data for anonymizing MDS data. Besides generating O/D pairs (flows), other geotemporal filtered metrics are generated, such as trip volume, availability, on-street, drop-offs, pick-ups, and more general metrics, such as vehicle utilization and average trip duration. Contrary to Dockless Open Data where a user has to execute the anonymization steps in a database, Mobility Metrics is a tool where only configuration from the user is required and outputs a machine-readable dataset in JavaScript Object Notation (JSON) format, and a dashboard that provides visualizations. The dashboard is constructed by embedding the JSON dataset into an HTML template. For geotemporal metrics, the following k-anonymity generalization process is implemented:

1. Time binning: the start timestamp of a trip is grouped into bins of 15 minutes, one hour, and one day
2. Geographic binning depends on the type of metric that is measured:
  For non-flows: geographic binning is done with H3 hexagons, a hexagon-based geospatial indexing system, at resolution 9 (Fig. 2a), or with road segments (Fig. 2b). Depending on the type of metric, one or more bins are selected. For example, the trip volume metric selects all the hexagons and road segments matching the trip’s geometry.
  For flows: first, the start and end locations of an O/D pair are binned into matching H3 hexagons. The centroid of the hexagon is then used (Fig. 2c).
3. Removing sparse bins: bins with less than three O/D pairs ($k=2$) are removed.

This approach uses geotemporal bins containing at least three counts ($k=2$). A user can increment *k* with the ‘privacyMinimum’ parameter to increase the rider’s privacy.

*Telraam* This citizen-science project originates from the city of Leuven (Belgium) and allows citizens to monitor the crowdedness in their street by installing a camera on their window [10]. Vehicle detections are sent to a central API and are anonymized with the following steps:

1. Time binning: the timestamp of a vehicle detection is grouped into bins of one hour, and one day.
2. Geographic binning: vehicle detections are binned per road segment to protect the citizen’s residence location.

Notice that k-anonymity generalization is applied with $k=0$, thus bins containing one vehicle count can be created. According to Telraam, two requirements must be fulfilled to allow reidentification. First, a unique vehicle needs to be detected at a certain place and time, and second, that unique behaviour needs to happen systematically. In other words, a pattern can be recognized. Experiments from Telraam [10] showed that no pattern could be retrieved from bins containing one vehicle owing to the coarse-grained time binning per hour. As a result, a third step of removing sparse bins is deemed unnecessary.

We have shown how k-anonymization is currently used in three mobility related studies. However, these studies do not consider ANPR data as data source and do not offer semantic data. Therefore, in the section that follows, we similarly describe the design choices we made in our method to anonymize ANPR data in correspondence with the start of the art.

## Method

To research whether anonymized ANPR data can still provide useful visualizations for local decision making, we developed the ANPR Metrics tool to generate anonymized data and dashboards from ANPR data. The tool is written in Javascript and available as open source^1^ . In this section, we provide an overview of its design: how the data flows when maintainers of ANPR data (police departments, cities, technology vendors) would use the ANPR Metrics Tool, and which anonymization techniques and semantic data specifications are used. The next section will provide more in-depth knowledge how the different specifications and visualizations weave together by applying the tool in a case study for the city of Kortrijk.

### Data flow

**Fig. 3:**
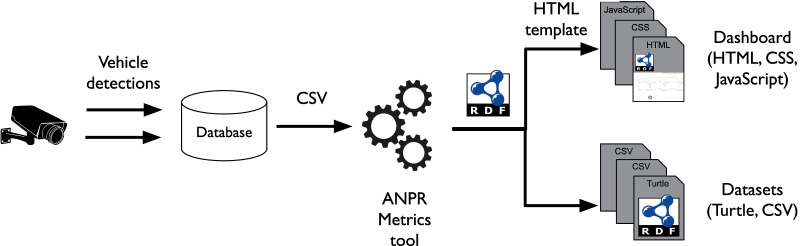
ANPR Metrics tool generates a dashboard and datasets from ANPR dataset

Figure 3 visualizes the data flow when using the ANPR Metrics tool. On the left in the diagram, a camera sends live vehicle detections to a vendor’s database. An export is made from this database with the non-proprietary Comma Separated Value (CSV) format which contains the columns mentioned in Table  1. Similar to the Mobility Metrics tool [5], the ANPR Metrics tool runs on a Command Line Interface (CLI). The privacy-sensitive CSV dataset is processed once, and is not saved persistently on disk. Otherwise, there would be a risk of spreading copies of the data. After anonymizing the data, the tool generates a user-friendly dashboard and machine-readable datasets in Turtle and CSV format. Turtle is a specific syntax for expressing RDF statements, all of which contain nodes linked with each other through relations and therefore represent a knowledge graph. A benefit of using a knowledge graph is that multiple features can be described in one file whereas a CSV file is limited due to its table structure.

The dashboard contains three documents: an HTML document that can be viewed in a browser, a CSS document to create the dashboard’s styling, and a JavaScript document that processes all the data and generates visualizations. Unlike most Web applications, there is no back end server involved to provide the dashboard with data, because the knowledge graph described in the Turtle file is embedded inside the HTML document. As a result, policy-makers can easily share the dashboard by exchanging these three documents and start exploring the dashboard in a browser by double-clicking the HTML document. The displayed visualizations will be further discussed in Sect. 5.1.

**Table 1:** CSV structure of ANPR dataset

Column name	Description
Plate	The license plate of a vehicle
Latitude	Latitude of the point location of the ANPR camera
Longitude	Longitude of the point location of the ANPR camera
TimeStamp	When the vehicle is detected
DeviceId	Identifier of the camera
Name	Label of the ANPR camera (e.g. the adjacent street name)

### Anonymization of ANPR data

Anonymization is performed, similar to the state of the art (Sect. 3), with k-anonymity generalization to avoid the possibility of individuals being identified. k-anonymity focuses on the release of datasets [18] while differential privacy is fit for query-response services where the output of a query is insensitive to the presence or absence of any individual in a dataset [30]. The anonymization process of ANPR Metrics works as follows:

1. Time binning: the timestamp of vehicle detection is grouped into bins of one hour, and one day.
2. Geographic binning depends on the type of metric that is measured:
  For non-flows: geographic binning is applied to the location of the ANPR camera where the vehicle has been detected.
  For flows: the start and end locations of the ANPR camera pair are used.
3. Removing sparse bins: bins containing less than ten vehicles ($k=9$) are removed.

This process is performed when generating anonymized data that pertain to geotemporal dimensions. Concretely, the ANPR Metrics tooling performs the process for the hourly and daily behavior and flows visualizations, which will be discussed in depth in Sect. 5. Time binning is performed per hour and per day, similar to the state of the art (Table 2). It is arguable to create time bins of 15 mins. However, the visualizations that are generated (Sect. 5) do not require smaller time bins. Future use cases with the data will have to indicate that such time binning size is desirable. In contrast with MDS trip data, which has a wide coverage of locations, ANPR camera locations are already a limited set. Therefore, geographic binning uses the location of the cameras without transformation. Moreover, referring to the accompanying road segment like Telraam is not required because an ANPR camera is part of the public domain and thus not privacy-sensitive information.

**Table 2:** Comparison of ANPR Metrics with the state of the art

	Mobility metrics	Dockless open data	Telraam	ANPR metrics
Geographic binning	Per road segment and between two road segments	Per road segment and between two road segments	Per road segment	Per point location and between two point locations
Time binning	15 min, hour and day	15 min, hour and day	hour and day	hour and day
k-anonymity generalization	2	4	0	9
Geographic fuzzing when below k	No	Yes	No	No
Format	JSON	CSV	JSON	RDF (Turtle)

The ANPR Metrics tool provides an option to indicate the minimum number of vehicles *k* per bin. By defining *k*, only observations with at least *k* vehicle counts will be published to prevent reidentifying an observation to one individual. Table 2 shows that Mobility Metrics and Dockless Open Data require at least three and five vehicle counts per bin respectively, while Telraam works for a minimum of one per hour. Our tool uses by default ten vehicle counts as minimum, which is double the threshold of the state of the art. Dropping small counts with a threshold mechanism is also done in Facebook’s Disaster Maps anonymization process [31]. Although no exact number is given, Kishore et al. [32] mention that bins with fewer than five to twenty users should not be shared with external parties. This way, decision-makers will only monitor observations that generate urban bustle instead of observations on an individual level. Having a minimum number of vehicles per bin higher than one is also necessary to prevent detections of patterns across datasets. For bins with only one geographic location, which is the case with Telraam (Sect. 3), observations with a unique vehicle count do not allow reidentification when no patterns can be detected. However, new patterns can be detected when combining datasets (inductive loop trace detectors [7], camera detections, mobile phone usage...), fulfilling the second requirement of Telraam. Therefore, the ANPR Metrics tool uses the same minimum number of vehicles for flows and non-flows.

De Montjoye [33] showed that human mobility traces are highly unique and that by knowing two geotemporal points approximately half of the traces can be uniquely identified. However, we argue that this result is not applicable to our proposed anonymization method. In his research [33], a pseudonymized mobile phone dataset *D* is used where every user has one trace *T* of geotemporal points. Pseudonymization is the process of replacing personally identifiable information with artificial identifiers, or pseudonyms [34]. The author states that the risk that *T* can be reidentified is related with a trace’s uniqueness *E*. Therefore, a scenario of a brute force characterization attack is considered where an adversary knows *Ip*, a set of p geotemporal points of an individual, and searches in *D* a subset of the traces, $S(I_p)$, that matches the *p* points composing $I_p$. When only one trace matches these points ($|S(I_p)| = 1$), the trace can be deducted to the user’s identity. The anonymization process of ANPR Metrics, but also Mobility Metrics and Dockless Open Data, generates traces of maximum 2 geotemporal points, which implies that a brute force characterization attack is only useful when an adversary already knows one geotemporal point ($p=1$) and finds a unique trace that matches the other point. Although unique traces are not generated due to k-anonymity generalization ($|S(I_p)| \ge k$), location privacy [19] is still an issue, because the observation can be linked to the user identity. Therefore, we advise to limit the information an adversary can infer of an individual by publishing traces (flows) of maximum two points. This type of attack is not useful for traces that contain one point (non-flows), such as traffic passersby at a specific location, because no new geotemporal points can be discovered of an individual.

### Data specifications used to model statistical data

As semantic interoperability is an essential aspect of a smart city’s Digital Twin, the knowledge graph generated by the ANPR Metrics tool needs to align with semantic specifications. Seven specifications are implemented in the tooling, which are based on the Flemish standardization programme^2^ Open Standards for Linked Organizations (OSLO) [22]. First, we will describe how we applied the Semantic Sensor Network (SSN) standard. Then, we will briefly describe the Core Location Vocabulary, GeoSPARQL, OWL-Time, OSLO Infrastructure Parts, City of Things Vocabulary and Fiware Traffic Flow Observed (Table 3).

**Table 3:** The base URI of a data specification is shortened with a prefix

Data standard	Prefix	Base URI
Semantic Sensor Network	ssn	http://www.w3.org/ns/ssn/
Sensor, Observation, Sample, and Actuator ontology	sosa	http://www.w3.org/ns/sosa/
City of Things	cot	https://w3id.org/cot#
OSLO Infrastructure	oslo-infra	https://wegenenverkeer.data.vlaanderen.be/ns/onderdeel#
NGSI-LD core metadata model	ngsi-ld	https://uri.etsi.org/ngsi-ld/
Traffic Flow Observed	fiware	https://uri.fiware.org/ns/data-models#
ISA Location Core Vocabulary	locn	http://www.w3.org/ns/locn#
OGC GeoSPARQL	geosparql	http://www.opengis.net/ont/geosparql#
OWL-Time	time	http://www.w3.org/2006/time#

*Semantic sensor network ontology* is a W3C standard for describing sensors and their observations, the involved procedures, the studied features of interest, the samples used to do so, and the observed properties, as well as actuators [35]. It is segmented into multiple modules, each providing classes and properties for a specific use case. The Sensor, Observation, Sample and Actuator (SOSA) ontology is used to provide the most common classes and properties across SSN modules. The *Observation* module allows to describe what, how, and when something is measured, and which sensor performed the observation. Applying SSN to raw sensor data, such as the output from an ANPR camera, is straightforward: an observation (*Observation*) originates from an ANPR camera (*Sensor*), which measures the number of vehicles (*ObservableProperty*) across a road segment (*Feature of Interest*) at a particular moment in time (*phenomenonTime*). Each time a vehicle passes a camera, a new observation can be created containing one vehicle (Result) and the time the vehicle passed by (*phenomenonTime*). In an alternative use case, SSN can also be applied to describe the ANPR Metrics tool’s outcome, also referred to as the ANPR statistical data. The tool then becomes the *Sensor* and the phenomenon time becomes a time interval instead of a timestamp. SSN does not define domain-specific properties to model the location, time, or what is observed. Therefore, we use other data specifications to fill these gaps.

*Core location vocabulary* is one of the core standards of the European ISA programme [21] to facilitate the interoperability of spatial information of public administrations^3^. The standard provides a minimum set of classes and properties for describing any place in terms of its name, address or geometry. A major advantage of the standard is its adoption in Flanders by OSLO, the region where we tested the ANPR Metrics tool. It is therefore an ideal candidate to describe, for example, an ANPR camera’s geometry.

*GeoSPARQL* is a standard from the Open Geospatial Consortium (OGC) for representing spatial information with RDF^4^. It also extends the SPARQL querying language for querying spatial information. Although the Core Location vocabulary allows describing the geometry of an entity, it does not require a specific encoding mechanism. Therefore, GeoSPARQL is used to encode geometry with Well-Known Text (WKT) literals specifically.

*OWL-time* or Time Ontology in OWL is a joint OGC-W3C [25] candidate standard to describe the temporal information of a resource^5^. Its core class is TemporalEntity providing properties to describe the begin and end time instant of a resource. Two subclasses from this core class are defined: Instant and Interval. They respectively describe a specific point in time and things with extent. With this standard, the temporal properties of SSN observations are described.

*OSLO infrastructure parts vocabulary* focuses on the description of physical objects that are part of the road structure, road appurtenances or infrastructure in a broad sense. A part is a uniquely identifiable component that makes up the (road) infrastructure. This vocabulary is created by the Flemish agency for roads and traffic (AWV) and is governed by the OSLO standardization program. We will explain later in this study how ANPR cameras are classified using this vocabulary.

*City of things vocabulary* (abbreviated CoT) is a vocabulary [36] providing a shared conceptual framework for smart city concepts with a focus on data exchange. CoT extends SSN with among others observable properties and features of interests. It is important for future applications that the statistical data generated by ANPR cameras use properties as specific as possible. For example, the property cot:passedByVehiclesCount is defined with ”The number of vehicles that are counted at a certain location, e.g. where a camera is observing.” and extends M3-lite, a taxonomy for, among others, crowd mobility [11] by adding a broader match relation with m3-lite:Count. CoT is currently not a standard, but can serve as input for a future standardization trajectory.

*Traffic flow observed* Smart Data Models (SDM)^6^, a global program led by among others Future Internet Ware (FIWARE), provides a set of vocabularies targeting smart city domains (e.g. street lighting, Smart Agrifood, waste management, transportation...). More specifically, the Traffic Flow Observed vocabulary allows describing traffic flow conditions at a certain place and time. Traffic intensities at one camera location can be described, but also between multiple ANPR cameras. This vocabulary extends the NGSI-LD metadata model [20], an RDF-based property graph data model for context information modelling.

In the next section, we will describe with data from the city of Kortrijk how each of the above data specifications is used and which visualizations can be generated on top of the data.

## Case study with ANPR data of Kortrijk

Several visualizations providing insights into the movement of vehicle categories (passenger vehicles, ligth-goods vehicles, heavy-goods vehicles) with ANPR data have been designed and tested in Mechelen (Belgium) [7]. The authors claim that the data are anonymized by replacing license plates with artifical identifiers, or pseudonyms, every week. However, replacing identifiers from data does not ensure that the remaining information is no longer identifiable [33, 37, 38]. In this section, we want to extend the related work of Mechelen by producing similar visualizations using our anonymization approach and validate whether they still contribute for policy-making.

### Dashboard and data model

**Fig. 4:**
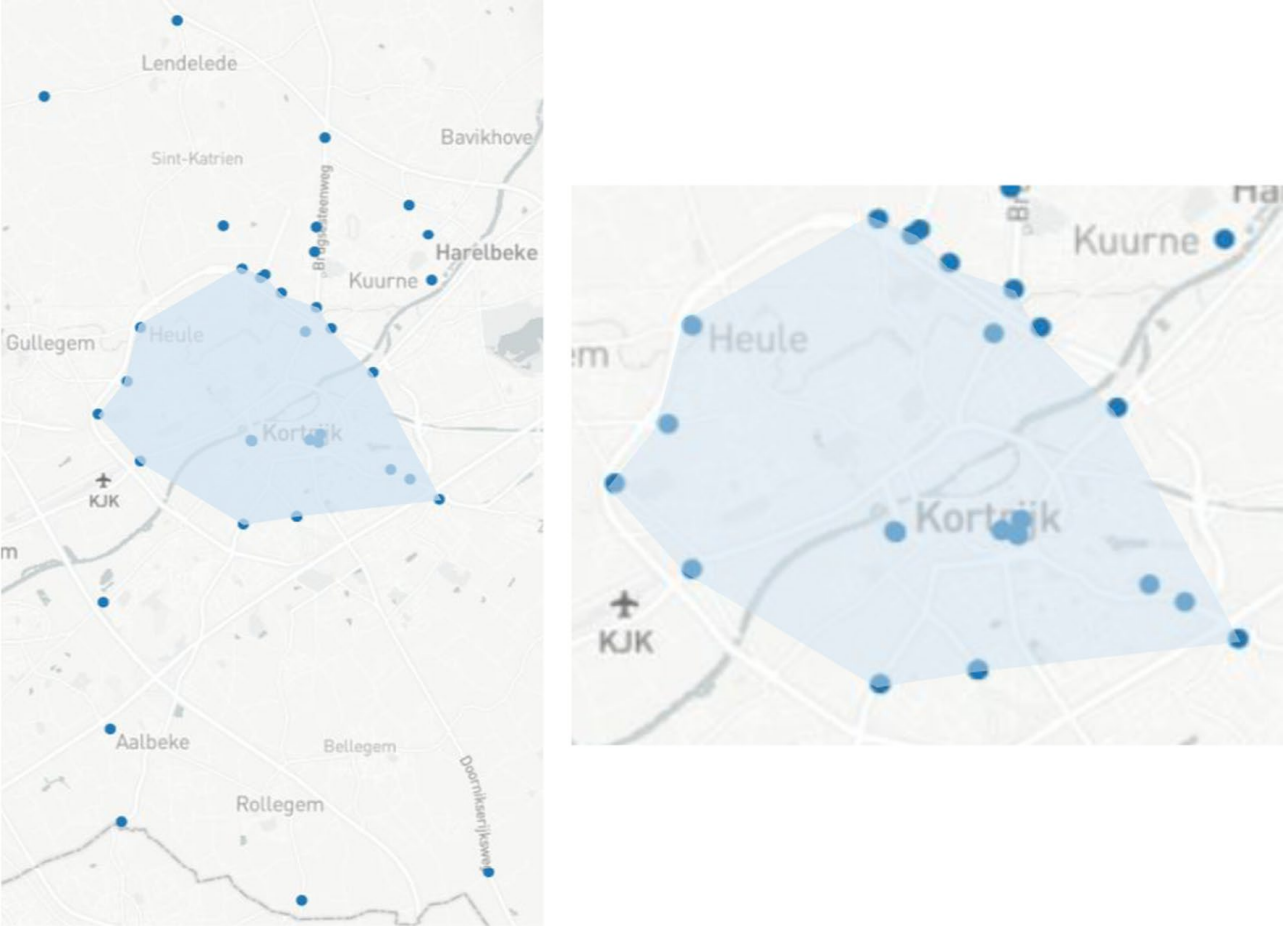
Map of ANPR cameras in Kortrijk with the city center displayed in more detail

ANPR data was obtained from Kortrijk (4th February until 5th March 2020) to evaluate the ANPR Metrics tool. This dataset contains vehicle detections from 43 cameras of which 32 are located in streets and 11 in parking facilities. Figure 4 shows that most cameras are located around the city center and that some cameras are located in the northern and southern municipalities. A dashboard is generated containing the embedded knowledge graph, which is made of a group of RDF statements serialized in Turtle format (Sect. 4.1). We will discuss below for every visualization which data specifications the ANPR Metrics tool uses and visualize the corresponding RDF statements of the knowledge graph for better readability. For generated objects that don’t have an existing identifier (URI), the Flemish URI standard is used [39]. URIs are constructed using following template: https://domain/type/concept(/reference)*. For example, *https://mycity.org/id/camera/123* illustrates an ANPR camera’s URI where *mycity.org* is the domain name of the city, *id* (type) is an identifier of the entity in the real world, *camera* (concept) is the category of the object, and *123* (reference) is a unique reference to this camera. Notice that the asterix in the template allows multiple references to be concatenated at the end of the URI. This will be used when identifying observations. In our dataset, we use *https://example.org/id/* as base URI with prefix ’my’, although the publisher should change this base URI to their domain. The raw ANPR dataset contains more than four million vehicle detections and is converted into 87k anonymized observations with the ANPR Metrics tool (Table 4). There are 7 classes (oslo-infra:ANPRCamera, locn:Geometry, sosa:Observation, time:Interval, time:Instant, cot:Flow, cot:AggregatedFlowCount) and 15 properties (rdfs:label, rdf:type, locn:geometry, geosparql:asWKT, cot:aggregationPeriod, sosa:phenomenonTime, time:hasBeginning, time:hasEnd, time:inXSDDateTimeStamp, sosa:hasFeatureOfInterest, sosa:observedProperty, sosa:hasSimpleResult, cot:numberOfSeconds, cot:usingFunction, cot:contains) used to create visualizations.

**Table 4:** Dataset key statistics

Category	Resource
Nr. of ANPR cameras	43
Nr. of raw vehicle detections	4034k
Nr. of SSN observations	87k
Total nr. of triples	1188k
Nr. of classes	7
Nr. of properties	15

**Fig. 5:**
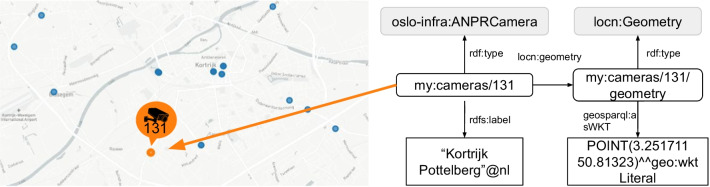
Overview of cameras in Kortrijk with camera 131 selected

*Camera overview* The first visualization of the dashboard is an overview of ANPR cameras where the user can select a camera for more details (Fig. 4). On Fig. 5, camera with local identifier ‘131’ is selected in orange. On the right-side of the figure, we see that the selected camera has my:cameras/131 as an identifier on the Web, and is an instance of an ANPRCamera. The term ANPRCamera is defined in the OSLO vocabulary for infrastructure parts, drawn up by the Flemish Agency for Roads and Traffic (AWV). As a result of using this agreed upon term, this camera is now interoperable with other cities in its region. Lastly, the camera is located in Kortrijk Pottelberg and uses the core location vocabulary to define its geographical position. The point location is formatted using the Well-known text (WKT) format for geometry.

**Fig. 6:**
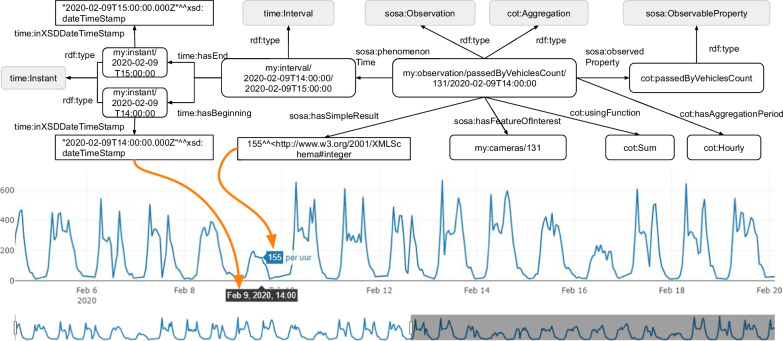
Hourly and daily behavior visualization. 155 vehicles passed camera 131 between 2 pm and 3 pm on Tuesday 9th February 2020

*Hourly and daily behavior* Traffic density fluctuates during the day according to activities happening in the city. The density fluctuation at the height of the selected camera from the camera overview is shown in Fig. 6 by plotting the number of vehicles that passed per hour. The user can hover over the chart and see that, for example, there passed 155 vehicles on Sunday 9th February in the afternoon, which is remarkably lower than on weekdays and Saturdays. This information is captured in the knowledge graph in three parts. On the top-left side of Fig. 6, the Time ontology is used to create an interval, which has a beginning and ending timestamp. Next, a sosa:Observation is defined for this particular data point on the chart that connects the time interval with the result (155) and what is observed (count of passed by vehicles). Third, the City of Things vocabulary is used to specify this observation as an aggregation by taking the sum of ANPR counts (*cot:Sum*) over hourly bins (*cot:Hourly*).

**Fig. 7:**
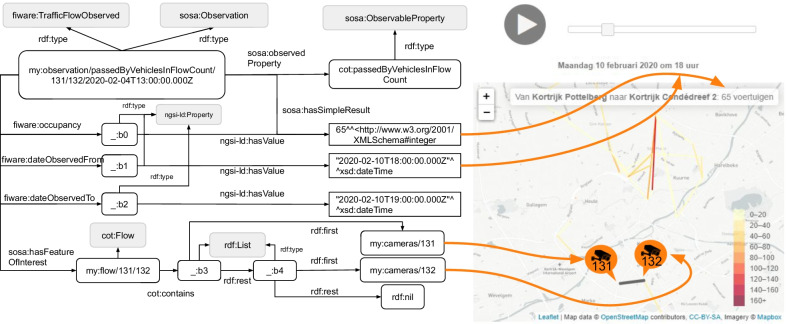
Flows visualization. There are 65 vehicles counted between Pottelberg and Condédreef indicating urban bustle

*Flows* Fig. 7 shows the number of vehicles per hour between two cameras, also called origin/destination (O/D) pairs (Sect. 3). Because of k-anonymity generalization of 9, flows contain minimally 10 vehicle counts. In contrast with the dashboards of Mobility Metrics and Dockless Open Data, a time slider is added to dynamically view the evolution of traffic between cameras. We can see on the figure that 65 vehicles went from Pottelberg (camera 131) to Condédreef (camera 132) at 18h on the 10th February. The color legend indicates that streets around this flow experience a fair amount of urban bustle during the peak-hours. This O/D pair will be further discussed in Sect. 5.2.

The knowledge graph for flows (Fig. 7) can be constructed using two data specifications in particular. First, SSN/SOSA allows to describe the observation of how many vehicles are counted between two cameras with *cot:passedByVehiclesInFlowCount*. To indicate which camera was the origin and destination of the flow, we use specialized terms of the CoT vocabulary: *cot:Flow* and *cot:contains*. The feature of interest is a flow, which contains an ordered group of cameras. *rdf:List* is used to express ordening through a combination of *rdf:first* and *rdf:rest* relations. Second, the Traffic Flow Observed specification can be used, which provides properties to describe the time interval (*fiware:dateObservedFrom*, *fiware:dateObservedTo*) and what the occupancy of the flow is (*fiware:occupancy*). In contrast with the SSN/SOSA approach, Traffic Flow Observed does not allow indicating the entities of a flow, but uses a *ngsi-ld:location* property to describe the flow’s geometry in GeoJSON format. Finally, the time interval description of flows is similar to the hourly behaviour visualization (Fig. 6).

**Fig. 8:**
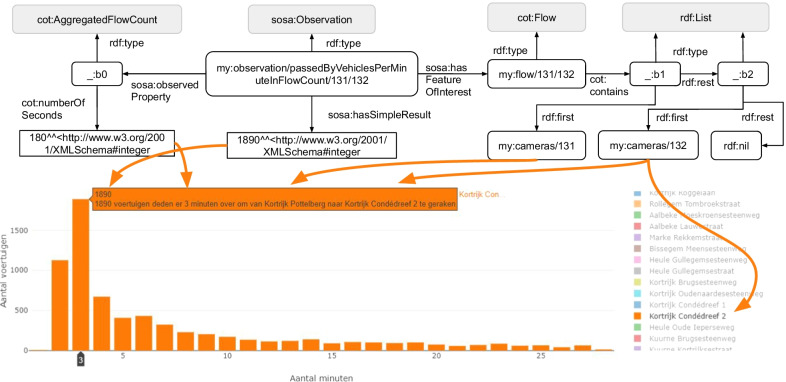
Travel time estimation visualization. Distribution of the number of minutes that is needed to travel between two ANPR cameras

*Travel time estimation* By visualizing how many vehicles took a certain amount of minutes to travel between two cameras, policy-makers can quickly see how long a flow usually takes. As origin is the camera from the camera overview used. On the right-side of Fig. 8, a user can select a destination camera from a list. In this example, the most occurring travel time between Pottelberg to Condédreef 2 is 3 minutes and is performed by 1890 vehicles in the dataset. These observations are generated over all days in the dataset, thus time binning is not performed. According to Bertini [40], ANPR data allow to accurately estimate the real-time travel time of a trip. However, accurate estimates require clustering travel times in time [41], such as working days versus weekends. Notwithstanding this limitation, Fig. 8 already gives a general overview of the most common travel times between cameras. The knowledge graph uses SSN/SOSA, similar to the flows visualization, to describe the number of vehicles per O/D pair. Next to returning how many vehicles have been counted, a property is required to describe the time that was needed by these vehicles. This property is added to *cot:AggregatedFlowCount*, which is a subclass of *sosa:ObservablePropery* to qualify the observable property. On Fig. 8, we see that the flow count is aggregated over vehicles that needed 3 minutes (180 seconds) to traverse the O/D pair.

**Fig. 9:**
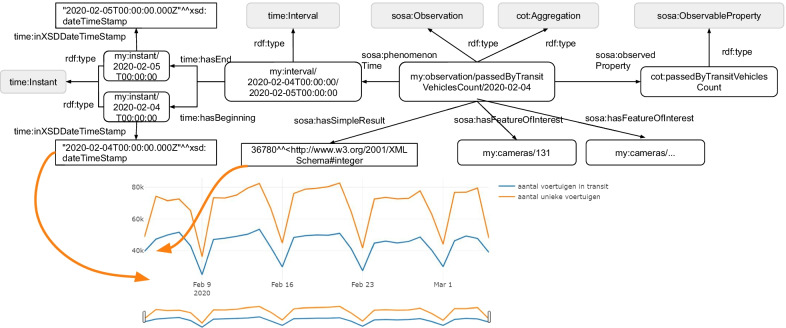
Unique versus in transit vehicles visualization. More than half of the detected vehicles are in transit

*Unique versus in transit vehicles* An important visualization for city administrations is the passersby that are being made through the city. In the smart cities “definition manual” for urban bustle [42], an “in transit” profile is proposed for determining whether a person is passing by and is not lingering or staying in the city. We applied this for vehicles instead of people, and added a second criterion to decide when a vehicle is in transit. First, the vehicle cannot be detected in the city for longer than an hour, and second, that vehicle may only be detected once per ANPR camera. To put in transit vehicles in perspective, the number of unique vehicles is also displayed on Fig. 9. We see that more than half of the vehicles are in transit. However, all ANPR cameras from the dataset are considered. To only consider cameras in a certain area, such as ring roads or approach roads, then the raw ANPR dataset needs to be filtered before using the ANPR Metrics tool.

The knowledge graph description of vehicles in transit is also demonstrated in Fig. 9. The SSN/SOSA observation is an aggregation of vehicles per day and is specified with the *cot:passedByTransitVehiclesCount* observed property. An ordered list of cameras is not required. Consequently, all cameras within the ANPR dataset are listed with the *sosa:hasFeatureOfInterest* relation.

### Validation with mobility expert

The mobility department of Kortrijk received in September 2020 the dashboard with above mentioned visualizations. In the next paragraphs, we will summarize their feedback and insights.

Currently, Kortrijk manages six traffic analysis devices that are used on roads maintained by the city to measure speed, intensities, and nuisance of heavy traffic. However, these devices are limited in their possibilities: the road may be too wide, or two cyclists can be misdetected as one car. Due to the temporary nature of these devices, a limited number of roads can be measured at the same time and this for a relatively short period of time. ANPR cameras are different from the traffic analysis devices in a number of respects: wider roads can be observed, vehicles are always reliably detected, and the cameras are permanently available.

A sustainable comparison of intensities between similar streets, e.g. approach roads, at the same time and during the same long period becomes possible, which is not possible with the devices of Kortrijk. The cameras are installed at strategic traffic points that are relevant for police tasks, such as roads connecting two villages. For Kortrijk, the cameras at approach roads near the ring road (R8) towards the city center are of particular interest. These approach roads are not managed by the city itself, but by the Flemish government. This way, anonymized ANPR data does not only overcome technical barriers, but also organizational barriers. This allows answering policy questions, such as how global traffic volume in the city is evolving or how many vehicles are transiting the city center [42]. In light of COVID-19, questions were regularly asked to compare traffic volume before and during COVID-19, for example between September 2019 and September 2020, to analyze the impact of measurements against the spread of the coronavirus. Also, these traffic volumes are an unique opportunity for policy-makers to analyze the impact on air quality [43].

**Fig. 10:**
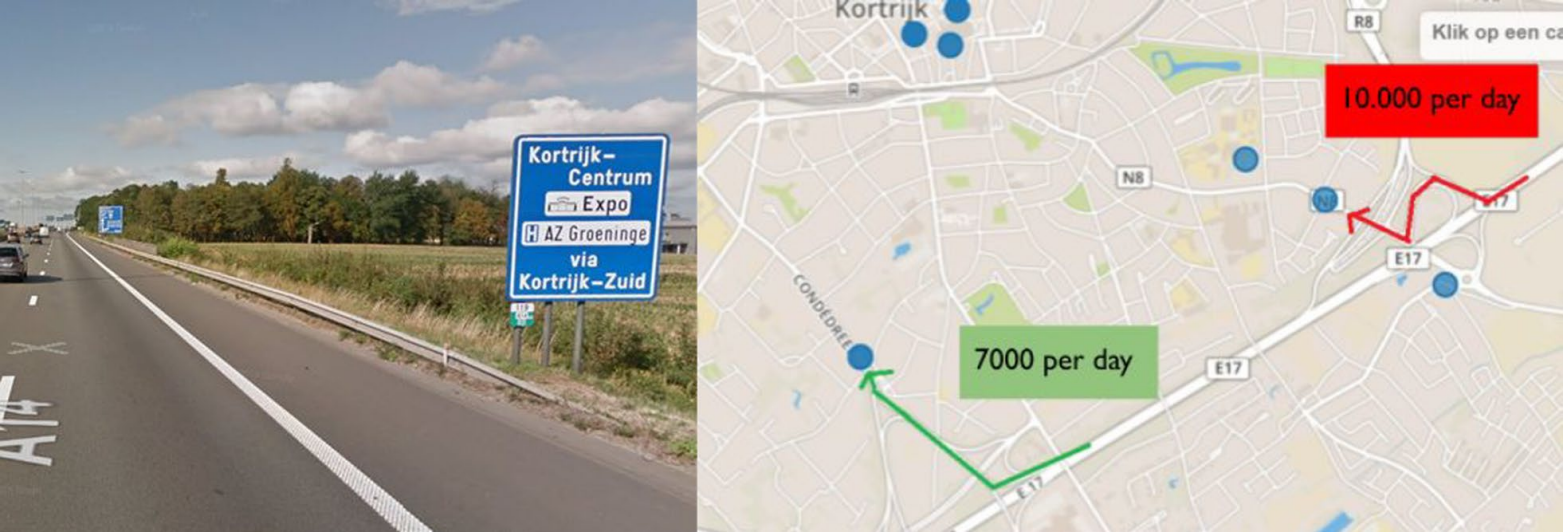
**a** Signposting guides traffic on the highway (E17) via Kortrijk South towards the city center. **b** Most of the traffic leaves the highway via Kortrijk East (red) instead of Kortrijk South (green)

Comparison of intensities can also indicate that there may be something wrong with signposting. This has been seen in the case of traffic going from the highway (E17) towards the center (Fig. 10a). In principle, traffic on the highway originating from the north-east is guided towards the center via Kortrijk South (Ei and Condédreef) and not via Kortrijk East (Oudenaardsesteenweg). When visualizing the daily behavior at these locations with the ANPR Metrics dashboard, peaks are observed above 10.000 vehicles in the densely built Oudenaardsesteenweg, while the Condédreef peaks reach only 7.000 (Fig. 10b). This undesired behavior needs to be addressed and followed up by the mobility department to examine whether this is a temporary phenomenon caused by road works, for example around the station (Panorama and Appel), by comparing traffic intensities with a period before these road works.

Statistical data from ANPR data are a valuable source of information for studies that aim to improve the mobility and livability of and around a certain part of a city. For example, the Flemish government is investigating with the K-R8 trajectory [44] how it can improve the livability in Kortrijk East and Hoog Kortrijk, both are city districts located around the ring road R8. To visualize the traffic density around these districts, the mobile traffic analysis devices of the city are used. However, enriching the current visualizations with the anonymized ANPR data would provide a more complete view on the traffic density in these districts. Also, having access to up-to-date traffic densities of the city would allow creating a feedback loop for policy-makers to adjust its mobility plan accordingly.

Another important mobility challenge that Kortrijk faces is investigating how much traffic takes shortcuts between two locations (origin and destination) instead of following the signposted route. More specifically, Kortrijk is interested in how many vehicles that depart from one side of the R8 ring road and arrive at the other side go through the city center instead of taking the ring road. However, the current coverage of cameras in the city center (Fig. 4) is insufficient to analyse the specific trajectory of these vehicles.

## Results and discussion

The case study with Kortrijk confirms that useful policy-making visualizations can be generated by anonymizing ANPR data first. Policy-makers can answer questions related to urban bustle like which locations in the city are busy, or how this complies with the cities’ mobility plan. Local authorities question whether time bins can be made smaller to, for example, bins of a quarter. This time precision would allow use cases such as measuring urban bustle at the opening and closing times of a school. We argue that, although anonymized quarterly time bins can be generated above a certain threshold, a coverage issue still exists with ANPR cameras to measure around specific locations. To date, research^7^,^8^ is undertaken on how urban bustle can be measured by combining high-level measurements from, among others, ANPR data with low-level measurements [42] originating from, for example, mobile traffic analysis devices. The generated visualizations of the ANPR Metrics tool are aligned with related work: the hourly and daily traffic behaviour are similar to Telraam [10] and Mechelen’s ANPR visualizations [7]. Also, the flows visualization is, except the timeline, similar with Mechelen, Dockless Open Data and Mobility Metrics [4, 5, 7]. Visualizing the distribution of travel times between camera pairs was chosen instead of the distribution of speed estimates, because the exact trajectory of a vehicle is not described in an ANPR dataset, thus only travel times can be accurately visualized. In future investigations, it might be possible to cluster travel times for more in depth analysis, such as clustering peak hours or holidays. Lastly, this study provides new insights into visualizing unique versus in transit vehicles to demonstrate profiling vehicles according to a smart city definition manual [7, 42].

An anonymization approach using k-anonymity generalization with time bins per hour and day is proposed. In contrast with state of the art techniques [4, 5, 10], we argue that fuzzing is futile for ANPR data. The generated time bins are dense, because the number of geographic locations is limited to the number of cameras and these cameras are installed at strategic, busy locations. However, it is necessary to protect a users’ privacy by removing sparse bins below a certain threshold of vehicles. The ANPR Metrics tool uses a threshold of 10 vehicles by default. This k-anonymity configuration is more than double of the other investigated techniques and the user is in control to increase this number. Notwithstanding the relatively limited number of related work [4, 5, 10, 31, 32], this work offers valuable insights into aggregating ANPR data in bins for non-flows, and flows containing two location points. A natural progression of this work is to analyse the k-anonymization method for higher dimensional ANPR data. Related work [7] used more vehicle attributes, such as emission norms and vehicle categories. Achieving differential privacy, which is more suited for high-dimensional data, via a t-closeness extension of k-anonymization [18] can be an interesting next step to publish these datasets.

The results of this study indicate which specifications are suited for the semantic description of the generated statistical data. This way, the data become self-describing and stimulate the creation of digital twin solution accelerators [12] that operate cross domains (air quality, sound prediction...). We aligned with nine existing specifications, such as the W3C standard for sensor measurements (SSN/SOSA [35]). More specific properties, such as referring to an origin or destination camera, were provided with the City of Things vocabulary. In Flanders, semantic data specifications are being developed led by the Open Standards for Linked Organizations (OSLO) standardization programme [22]. As a result, a term for identifying ANPR cameras could be reused from the OSLO Infrastructure Parts vocabulary. Based on this study, we suggest that OSLO creates an application profile for the exchange of anonymized mobility datasets. This way, a normative specification will be available for technology vendors indicating how they can be compliant with our approach. Also, local authorities can refer to this specification during public tendering enabling new datasets using semantic technologies at the source. This approach allows cities to comply with the tenth principle of the Open Data charter [3], which is signed by the 13 biggest cities in Flanders, and Brussels. This principle states that Linked Open Data, which uses the same principles as the semantic data specifications, must be used for new datasets, especially for authentic sources and datasets that will be widely shared.

## Conclusion

This study has shown that the anonymization approach k-anonymity, which is already adopted in other mobility related areas, is also usable for ANPR data. Related work in visualizing ANPR data for policy-making is extended by demonstrating how visualizations can still be created with anonymized instead of pseudonymized data. Also, the state of the art is improved by exploring how existing semantic data specifications can be used to describe the anonymized, statistical data. This way, the data can reach its full potential in, for example, digital twins.

The challenge now is to increase trust in publishing anonymized ANPR datasets [38]. Currently, in Flanders ANPR data and its derivatives that are maintained by police authorities cannot be reused for smart city purposes by law. Given our proposed anonymization technique for ANPR data is based on already performed work in the mobility domain, we believe that statistical data originating from ANPR cameras can be published similar to official statistical averages [45, 46]. This way, cities can increase transparency of the data that is collect from citizens on the public domain. Our validation with a mobility expert from the city of Kortrijk acknowledges that these statistical data are useful for cities in multiple use cases allowing them to optimize their policy-making with sustainable data sources.

As next step, we are currently advising a technology vendor operating in the smart region of Limburg in Flanders (S-Lim) by repeating this study for their cameras. With these kind of initiatives, we hope to inspire other cities and technology vendors to start implementing statistical ANPR data following semantic data specifications.

## Data Availability

Data and dashboard of the case study are available from the authors upon reasonable request and with permission of Kortrijk. The primary ANPR dataset and datasets generated and/or analyzed during the case study are not publicly available due to juridical restrictions of using this data, but the source code and an example dataset are available in the anpr-metrics repository: https://github.com/brechtvdv/anpr_metrics.

## Footnotes

1 https://github.com/brechtvdv/anpr-metrics.

2 https://data.vlaanderen.be.

3 https://joinup.ec.europa.eu/collection/semantic-interoperability-community-semic/solution/core-location-vocabulary.

4 https://www.ogc.org/standards/geosparql.

5 https://www.w3.org/TR/owl-time/.

6 https://smartdatamodels.org/.

7 https://www.imeccityofthings.be/en/projecten/cityflows.

8 https://www.brugge.be/vloed-voorspellingen-lokale-ondernemers-en-economie-voor-drukte.
